# User-Friendly and Responsive Electrochemical Detection Approach for Triclosan by Nano-Metal–Organic Framework

**DOI:** 10.3390/molecules29143298

**Published:** 2024-07-12

**Authors:** Xiaoyu Li, Gaocheng Zhang, Zareen Zuhra, Shengxiang Wang

**Affiliations:** 1 School of Bioengineering and Health, Wuhan Textile University, Wuhan 430200, China; 2 State Key Laboratory of New Textile Materials and Advanced Processing Technologies, Wuhan Textile University, Wuhan 430200, China; 3 School of Mathematical and Physical Sciences, Wuhan Textile University, Wuhan 430200, China

**Keywords:** metal–organic framework, simple detect, triclosan, environmental monitoring

## Abstract

Antimicrobial resistance poses a significant challenge to public health, and is worsened by the widespread misuse of antimicrobial agents such as triclosan (TCS) in personal care and household products. Leveraging the electrochemical reactivity of TCS’s phenolic hydroxyl group, this study investigates the electrochemical behavior of TCS on a Cu-based nano-metal–organic framework (Cu-BTC) surface. The synthesis of Cu-BTC via a room temperature solvent method, with triethylamine as a regulator, ensures uniform nanoparticle formation. The electrochemical properties of Cu-BTC and the signal enhancement mechanism are comprehensively examined. Utilizing the signal amplification effect of Cu-BTC, an electrochemical sensor for TCS detection is developed and optimized using response surface methodology. The resulting method offers a simple, rapid, and highly sensitive detection of TCS, with a linear range of 25–10,000 nM and a detection limit of 25 nM. This research highlights the potential of Cu-BTC as a promising material for electrochemical sensing applications, contributing to advancements in environmental monitoring and public health protection.

## 1. Introduction

Antimicrobial resistance constitutes resistance to antibiotics, antivirals, antiparasitic agents, and antifungals, presenting a formidable obstacle to the effective management of microbial infections and representing a paramount public health concern [1]. While inherent, antimicrobial resistance is significantly expedited by the widespread misapplication of antibiotics in both daily life and medical contexts [2,3]. Triclosan (TSC) is a broad-spectrum antimicrobial agent that is commonly added at concentrations in the range of 0.1% to 1% to a variety of household and personal care goods (including shoes, textiles, toothpaste, soaps, detergents, and cosmetics) in order to inhibit the growth of bacteria, and its use is reported over 750,000 tons annually [4,5]. However, recent studies have revealed that the misuse of this broad-spectrum antimicrobial agent has resulted in the widespread presence of low concentrations of TSC in domestic sewage, urban water supplies, and groundwater [6,7]. This not only facilitates the development of antimicrobial resistance but also has the potential to cause the breakdown and formation of hazardous compounds like dioxins and chlorophenols, resulting in significant detrimental effects on both ecosystems and human health [8,9,10]. Therefore, establishing a rapid, simple, and efficient method for detecting trichloromethane in the environment is crucial for environmental protection and the well-being of individuals. Various analytical techniques have been developed for detecting TCS, including high-performance liquid chromatography (LC) [11], liquid chromatography–mass spectroscopy (LCMS) [12,13] gas chromatography–mass spectrometry (GCMS) [14,15], and the chemiluminescence method [16]. Despite their benefits like low detection limit and accuracy, time-consuming steps, the heavy use of organic solvents, and intricate protocols are problems for many of these techniques.

Electrochemical techniques for TCS detection excel among these alternatives, offering exceptional sensitivity, selectivity, rapid response time, and operational simplicity [17]. The phenolic hydroxyl group in TCS exhibits excellent electrochemical reactivity. A notable oxidation current response signal is produced on the electrode surface when voltage is applied, originating from the irreversible electrochemical oxidation reaction of TCS [18]. By measuring the intensity of this oxidation current, it can be directly correlated with the concentration of TCS in the sample. In addition to the electrochemical activity of TCS, the magnitude of the response signal often depends on the electrode modification material of the electrochemical sensor. High-quality electrode modification materials can significantly enhance the response current, thereby improving detection sensitivity. The use of sensitive materials to modify the electrode has led to the reporting of several TCS electrochemical detection techniques, such as the use of metals [19], metal sulfides [20], MWCNT [21], graphene composites, etc. [22,23,24]. Electrochemical sensitivity, selectivity, affordability, and ease of preparation could all be further improved with the help of developments in electrode and nanomaterial technology.

A metal–organic framework (MOF) is a class of porous materials formed by the self-assembly of metal ions or metal clusters with organic ligands via coordination bonds [25]. They have attracted widespread attention due to their unique features, such as large surface area, high porosity, tunable pore structure, and surface functional groups, as well as abundant unsaturated metal active sites. MOFs have been widely applied in various fields including gas storage and separation [26], energy storage and conversion [27], and molecular recognition [28]. The excellent properties of MOFs suggest their potential as promising electrochemical sensing materials. The large surface area and high porosity of MOFs provide a significant active surface area, while the unsaturated coordination metal sites offer exposed catalytic active centers, thus enhancing the performance of electrochemical sensing [29,30]. Therefore, the utilization of MOFs for constructing electrochemical monitoring platforms holds great promise for the future. Therefore, the utilization of MOFs for constructing electrochemical monitoring platforms holds great promise for the future. Notably, several MOF-based electrochemical sensors for TSC have already demonstrated excellent detection sensitivity and potential for practical applications [31,32]. Cu-BTC, with its variable valence metal ion Cu^2+^, facilitates electron transfer between Cu(II)-BTC/Cu(I)-BTC under applied voltage, enhancing the electron gain and loss processes of the analyte which results in significant electrochemical sensing amplification [33]. Additionally, Cu-BTC can be synthesized under mild conditions at room temperature via an interface method, making it highly promising for TSC rapid electrochemical detection.

In this work, nano-Cu-BTC material was prepared using a solvent technique at room temperature. Triethylamine was added to control the rate of ligand deprotonation, which in turn controlled the synthesis of more homogenous Cu-BTC nanoparticles. A thorough analysis was undertaken to comprehensively characterize the acquired nano-Cu-BTC. This included a complete investigation into its electrochemical properties, as well as the electrochemical behavior of nano-Cu-BTC on the triclosan surface. Additionally, the study explored the mechanism behind signal amplification. Thus, by leveraging the signal amplification effect of nano-Cu-BTC, a triclosan electrochemical sensor was successfully developed. The experimental conditions were systematically optimized using response surface methodology, resulting in a simple, rapid, and highly sensitive triclosan detection method with a linear range of 25–10,000 nM and a detection limit of 25 nM.

## 2. Results

### 2.1. Structure and Morphology of Nano-Cu-BTC

**Figure 1:**
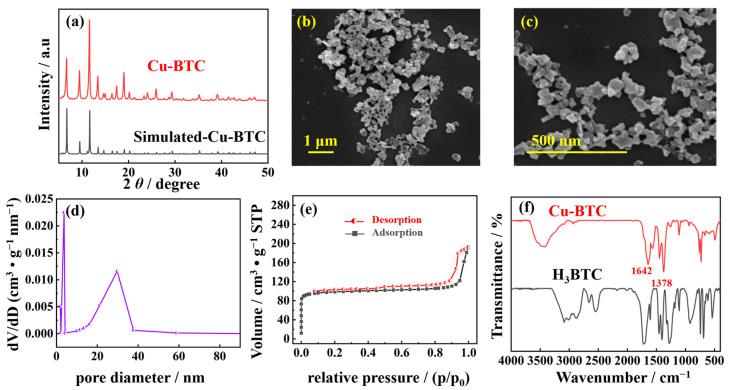
(**a**) XRD pattern of simulated and prepared Cu-BTC. (**b**,**c**) SEM images of nano-Cu-BTC. (**d**) Pore size distribution of nano-Cu-BTC. (**e**) N_2_ adsorption–desorption isotherms of nano-Cu-BTC. (**f**) FT-IR spectra of H_3_BTC and Cu-BTC.

The nano-Cu-BTC powder samples were analyzed for their crystal structure and composition using X-ray diffraction (XRD). Figure 1a demonstrates that the simulated standard XRD pattern of Cu-BTC and the XRD pattern of the synthesized Cu-BTC powder sample displayed corresponding characteristic peak positions, which aligns with previous research findings [34]. This confirms the effective synthesis of Cu-BTC. The average crystallite size of nano-Cu-BTC was calculated using the Scherrer equation: D = 0.9*λ*/(FWHM × cos*θ*), where *λ* is the X-ray wavelength (Cu-Ka radiation, 0.154 nm), FWHM is the full width at half maximum, and *θ* is the Bragg angle. The crystallite size of the Cu-BTC was determined to be 42.31 nm. The structure of the synthesized Cu-BTC was investigated using scanning electron microscopy (SEM). As seen in Figure 1b,c, the material had a non-uniform structure consisting of nanoparticles. The presence of triethylamine as a fundamental component in the MOF reaction is responsible for this irregular nanoparticle structure. Triethylamine speeds up the nucleation and growth rates of MOFs, as well as the coordination of metal centers and the rate at which organic ligands deprotonate. In contrast to the conventional block architectures at the micron scale, this nanoparticle structure is likely to be advantageous for the exposure of active sites and the improvement of reactivity. The surface area and pore size distribution of nano-Cu-BTC were determined using nitrogen adsorption–desorption isotherms, as shown in Figure 1d,e. According to the Brunauer–Emmett–Teller (BET) results, the specific surface area of the prepared Cu-BTC was determined to be 289 cm^2^ g^−1^, with a pore size centered around 20 nm. Figure 1f compares the Fourier-transform infrared (FT-IR) spectra of H_3_BTC and obtained Cu-BTC. The absorption peak of C-OH at 1278 cm^−1^ corresponding to the H_3_BTC ligand disappeared in the Cu-BTC spectrum. Additionally, two characteristic absorption peaks appeared at 1378 and 1642 cm^−1^ in nano-Cu-BTC, corresponding to the asymmetric and symmetric stretching vibrations of carboxyl groups, respectively. These changes in the infrared absorption peaks indicate the coordination of deprotonated H_3_BTC with Cu^2+^ centers, confirming effective coordination between Cu^2+^ and H_3_BTC [35].

### 2.2. Electrochemical Performance of Nano-Cu-BTC

**Figure 2:**
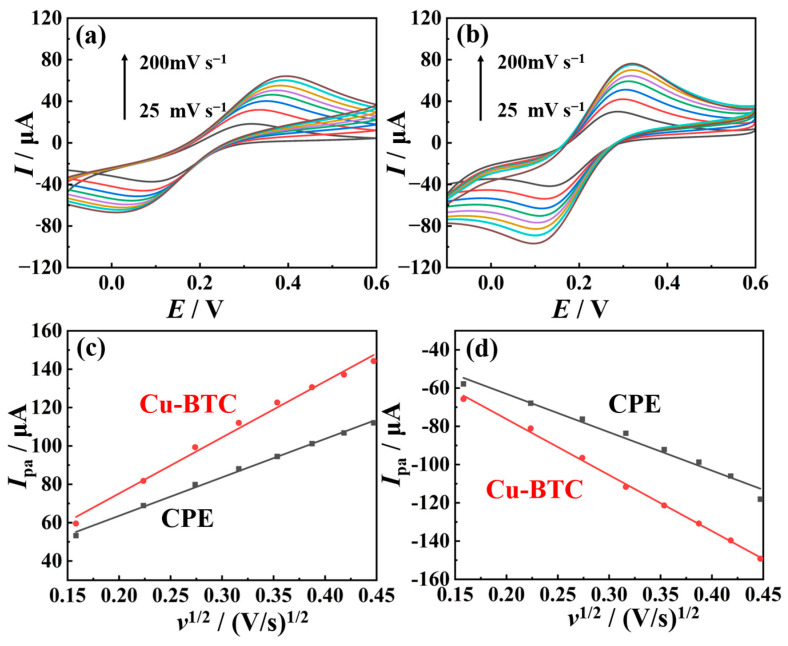
Cyclic voltammograms of CPE (**a**) and nano-Cu-BTC (**b**) in 0.1 M KCl solution containing 5 mM K_3_[Fe(CN)_6_] at scan rates of 25, 50, 75, 100, 150, 175, and 200 mV s^−1^. Linear relationship between *I*_pa_ (**c**) or *I*_pc_ (**d**) and square root of *v*.

The electrochemical response area of the nano-Cu-BTC-modified electrode and bare carbon paste electrode (CPE) were measured using cyclic voltammetry (CV) according to the Randles–Sevcik equation with K_3_[Fe(CN)_6_] as the probe [36]:

(1) $$I_{pa}=2.69\times 10^{5}AD^{1/2}n^{1/2}v^{1/2}C_{0}^{*}$$

where *I*_pa_ represents the oxidation peak current value, *A* is the electrochemical response area of the electrode, *D* is the diffusion coefficient, *n* and $C_{0}^{*}$ are the number of transferred electrons and the initial concentration of K_3_[Fe(CN)_6_], respectively, and *v* is the CV scan rate. In a 0.1 M KCl solution containing 5.0 mM K_3_[Fe(CN)_6_], CVs of different electrode surfaces were recorded at various scan rates (Figure 2a,b). Subsequently, the *I*_pa_ was plotted against the square root of the *v*, as shown in Figure 2c, and the slope of *I*_pa_ vs. *v*^1/2^ was used to calculate the electrochemical active area of different electrodes, which were determined to be 0.0253 cm^2^ (CPE) and 0.0323 cm^2^ (Cu-BTC). From the results, it is inferred that the introduction of nano-Cu-BTC can enhance the electrochemical active area of the electrode.

**Figure 3:**
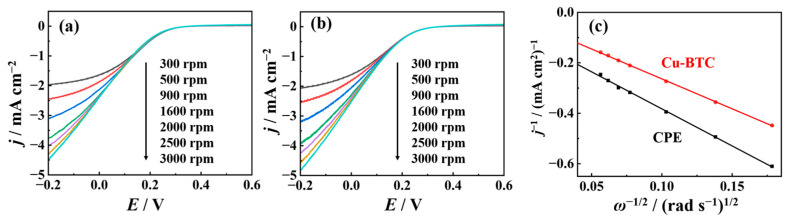
Linear sweep voltammograms of CPE (**a**) and nano-Cu-BTC (**b**) at various rotation speeds in 1 M KCl solution containing 5 mM K_3_Fe(CN)_6_, with scan rate of 100 mV s^−1^. (**c**) Koutecky–Levich plots for CPE and nano-Cu-BTC.

Linear sweep voltammograms of both CPE and nano-Cu-BTC were measured using a rotating disk electrode in a 0.1 M KCl solution containing 5 mM K_3_[Fe(CN)_6_], as shown in Figure 3a,b. The electron transfer rate constants were calculated based on the Koutecky–Levich equation [37]:

(2) $$\frac{1}{j}=\frac{1}{j_{L}}+\frac{1}{j_{K}}=\frac{1}{0.62nFc_{0}{\left(D_{0}\right)}^{\frac{2}{3}}v^{-\frac{1}{6}}\omega^{\frac{1}{2}}}+\frac{1}{nFkc_{0}}$$

Here, *j*, *j*_d_, and *j*_k_ represent the current density, the limiting diffusion current density, and the electron transfer current density (mA cm^−2^), respectively. *n* is the number of transferred electrons, *F* is the Faraday constant, *c*_0_ and *D*_0_ are the initial concentration and diffusion coefficient of K_3_Fe(CN)_6_, *ν* is the dynamic viscosity of the supporting electrolyte, *ω* is the angular velocity of the rotating disk electrode (rad s^−1^), and k is the electron transfer rate constant. As shown in Figure 3c, the calculated values of *k* on CPE and Cu-BTC/CPE were found to be 0.0116 cm s^−1^ and 0.0139 cm s^−1^, respectively. A higher *k* value indicates a stronger electrocatalytic capability of the electrode surface [38]. Therefore, the enriched variable-valence active metal centers in nano-Cu-BTC significantly enhance its electrocatalytic performance compared to CPE.

**Figure 4:**
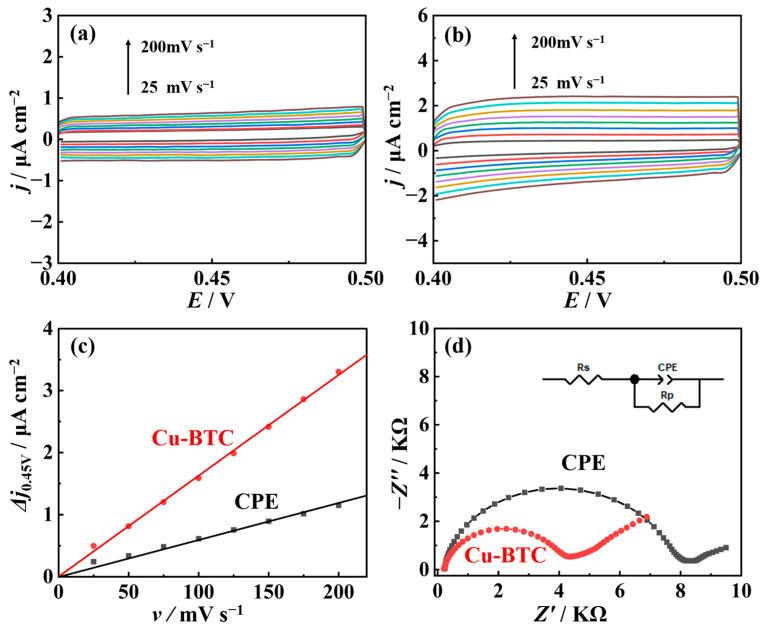
CVs of CPE (**a**) and nano-Cu-BTC (**b**) in 1 M KCl solution at scan rates, respectively. (**c**) Linear plot of current density at 0.45 V versus scan rate of CPE and nano-Cu-BTC. (**d**) Impedance spectra of CPE and nano-Cu-BTC in 1 M KCl solution containing K_3_Fe(CN)_6_/K_4_Fe(CN)_6_, with inset showing equivalent circuit used for fitting.

To further verify the influence of the electrochemical active area of nano-Cu-BTC, we demonstrated it through the calculation of specific capacitance. In 1.0 M KCl, cyclic voltammetry curves of CPE and nano-Cu-BTC at various scan rates (ν = 25, 50, 75, 100, 125, 150, 175, and 200 mV s^−1^) in the non-Faradaic region were plotted, as shown in Figure 4a,b. The difference in current density at 0.45 V (Δ*j* = j_anode_ − jc_athode_) relative to the scan rate was used to calculate the k value, whereas *C*_dl_ was calculated according to the following equation [39]:

(3) $$C_{dl}=\frac{1}{2}km^{-1}$$

where m is the reaction area of nano-Cu-BTC. The slopes of the lines allowed the calculation of *C*_dl_ for CPE and nano-Cu-BTC as 4.3 μF cm^−2^ and 31.6 μF cm^−2^, respectively (Figure 4c). Since the electrochemical active area is proportional to *C*_dl_, prepared nano-Cu-MOF can significantly enhance the electrochemical active area of the electrode. In order to further validate the enhanced electron transfer capability of nano-Cu-BTC, we investigated the electrochemical impedance behavior on the surfaces of both CPE and nano-Cu-BTC using K_3_Fe(CN)_6_/K_4_Fe(CN)_6_ as probe molecules. As shown in Figure 4d, a large diameter semicircle appeared on the CPE, while the semicircle diameter notably decreased on nano-Cu-BTC, indicating a significant reduction in interface impedance. According to the fitted equivalent circuit, the charge transfer resistances (*R*_ct_) of CPE and nano-Cu-BTC were determined to be 778 kΩ and 389 kΩ, respectively. The lower charge transfer resistance of nano-Cu-BTC suggests its ability to enhance electron transfer efficiency.

**Figure 5:**
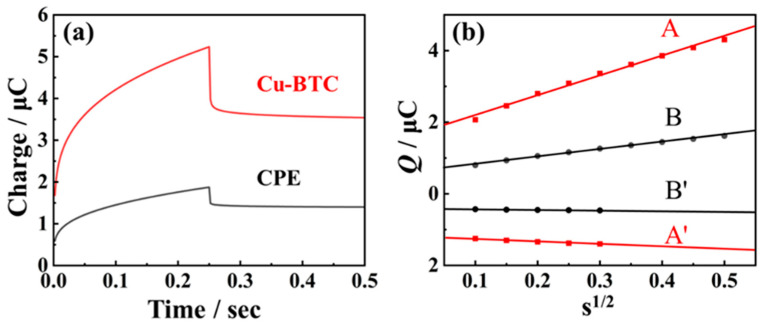
(**a**) Double potential step chronocoulometry curves of 10 μM TSC on CPE and nano-Cu-BTC. (**b**) Plots of *Q*_f_-t^1/2^ during the forward step on CPE (A), and nano-Cu-BTC (B), as well as *Q*_r_-*f*(t) during the reverse step on CPE (A′), and nano-Cu-BTC (B′).

To comprehensively elucidate the sensitization mechanism of nano-Cu-BTC, we employed a double-step chronoamperometry technique to investigate the electrochemical adsorption capability of nano-Cu-BTC. Within the potential range of 0.3–0.9 V, the charge-time (*Q*-*t*) curves for the forward adsorption (*Q*_f_)/reverse desorption (*Q*_r_) on both the CPE and nano-Cu-BTC surface were recorded in a phosphate buffer solution at pH 7.0 containing 10 μM TSC (Figure 5a). According to the Cottrell theory [40], the value of the total adsorbed charge *Q*_ads_ on different electrode surfaces can be calculated.

(4) $$Q_{f}=2nFAC*D^{1/2}\pi^{-1/2}t^{1/2}+Q_{dl}+Q_{ads}$$

(5) $$Q_{r}=2nFAC*D^{\frac{1}{2}}\pi^{-\frac{1}{2}}(\tau^{\frac{1}{2}}+(t-\tau {)}^{\frac{1}{2}}-t^{\frac{1}{2}})+Q_{dl}$$

*Q*_dl_ represents the double-layer charge, *Q*_ads_ represents the Faraday charge for the oxidation of adsorbate, *n* represents the number of electron transfers in the oxidation process, *F* is the Faraday constant, *A* is the electrode area, C* and *D* represent the diffusion coefficient and concentration of reactants respectively, *t* is time, and *τ* is the step time. The *Q*_ads_ value corresponds to the amount of substance participating in oxidation reactions adsorbed on the material surface, effectively reflecting the electrochemical adsorption capacity of the electrode material. It can be calculated that the adsorption amounts of trichloroethylene on the CPE and nano-Cu-BTC surfaces are 0.893 μC and 2.83 μC, respectively, indicating a stronger adsorption capacity of the prepared nano-Cu-BTC for TSC (Figure 5b). Based on this, it can be concluded that nano-Cu-BTC’s significant enrichment and electron transfer capabilities are mostly responsible for TSC’s oxidation enhancement, offering nano-Cu-BTC a stronger signal amplification current.

### 2.3. Mechanistic Study of TSC Oxidation Reaction

**Figure 6:**
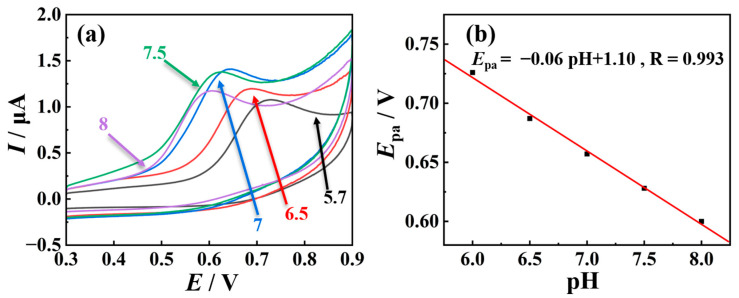
(**a**) CVs of 5 μM TSC in 0.1 M phosphate buffer solutions at different pH values on nano-Cu-BTC; (**b**) linear relationship between pH values and *E*_pa_ of TSC.

Further research on the signal amplification mechanism in electrode materials becomes feasible by enhancing the TSC interface oxidation process, which further makes it easier to study the interface reaction processes in TSC. Initially, the oxidation behavior of TSC on nano-Cu-BTC was compared using CV in phosphate buffer solutions with varying pH values. As depicted in Figure 6, the oxidation peak potential of TSC exhibits a progressive leftward shift with increasing pH, indicative of proton involvement in the oxidation process. Moreover, the slope of the *E*_pa_ versus pH curve approximates −0.06 V/pH, reminiscent of the Nernst equation, suggesting an equimolar participation of protons and electrons in the oxidation reaction of TSC. Furthermore, it is observed that as the pH ascends from 5.7 to 7, the oxidation peak current of TSC demonstrates a gradual augmentation. However, upon further increase in pH from 7 to 8, the oxidation peak current commences a gradual decline. Consequently, the oxidation signal of TSC attains its maximum at pH = 7.

**Figure 7:**
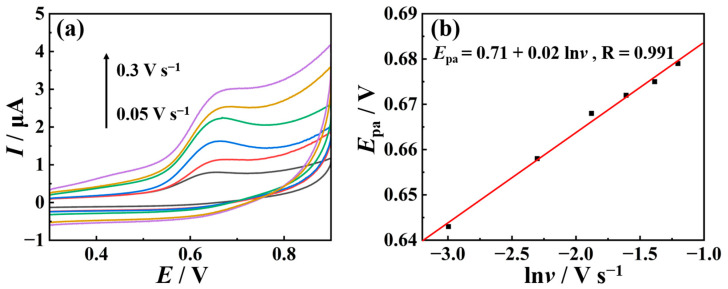
(**a**) CV curves of 5 μM TSC at pH 7.0 under different scan rates on nano-Cu-BTC. (**b**) *E*_pa_-ln*v* plot of TSC.

The electrochemical behavior of TSC on nano-Cu-BTC was investigated using CV at various scan rates (*v* = 50, 100, 150, 200, 250, and 300 mV s^−1^). As illustrated in Figure 7, with an increasing scan rate, the oxidation peak potential (*E*_pa_) of TSC gradually shifts towards the positive direction, indicating the irreversibility of the oxidation process of TSC. In addition, *E*_pa_ is directly proportional to the logarithm of the scanning rate (*v*), with a linear equation of *E*_pa_ = 0.71 + 0.02 ln*v*; the regression coefficient (R) is 0.991.

### 2.4. TSC Detection Method Based on Cu-BTC

In order to determine the optimal circumstances for TSC detection, the Design Expert 13 software was utilized. This software employs a response surface optimization approach within the Analysis module. The investigation focused on assessing the impacts of enrichment potential, Cu-BTC modification quantity, and enrichment time on the detection response signal of TSC. The experimental parameters were determined based on the experimental design supported by the Design Expert program, as outlined in Table 1. In addition, the oxidation current values of TSC were measured three times for each of the 17 prescribed conditions, and the resulting means were recorded.

**Figure 8:**
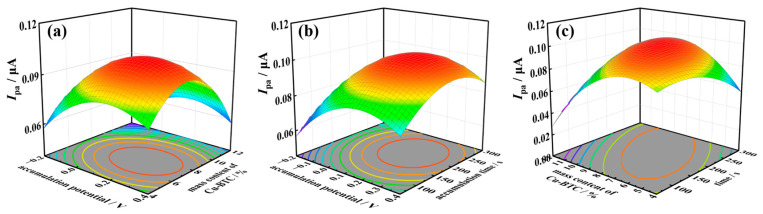
A 3D response surface and 2D contour plots for enrichment potential, nano-Cu-BTC modification amount, and enrichment time. (**a**) enrichment potential and nano-Cu-BTC modification amount, (**b**) enrichment potential and enrichment time, (**c**) nano-Cu-BTC modification amount and enrichment time.

Based on the experimental findings presented in Table 1, the optimal detection conditions were determined through software fitting. As shown in Figure 8, the optimal conditions are enrichment time: 224.78 s; enrichment potential: 0.2077 V; and modifier amount: 8.19%. Moreover, the *p*-values < 0.05 confirm the reliability of the model. Considering the specific experimental operating conditions, the determined parameters are set as follows: enrichment time: 225 s, enrichment potential: 0.2 V, and modifier amount: 8%. The peak current of TSC oxidation measured under these conditions shows no significant difference from the values obtained under the preset optimized conditions provided by the software, indicating the feasibility of the TSC detection parameters used.

**Figure 9:**
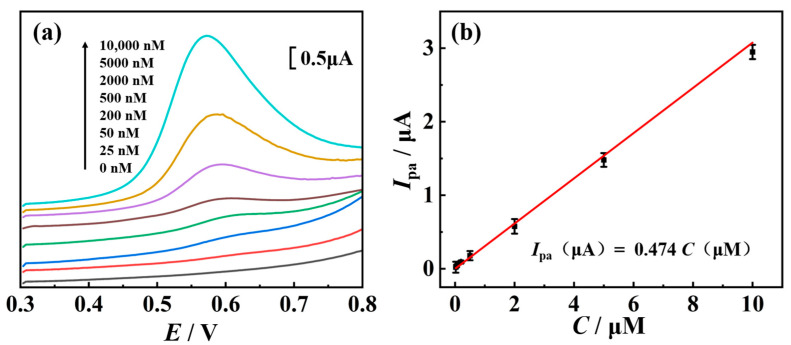
(**a**) DPV curves under different concentrations of TSC on nano-Cu-BTC. (**b**) Linear relationship between *C* and *I*_pa_ of TSC. Error bars represent standard deviation of three measurements.

Subsequently, under the optimal conditions obtained through response surface optimization, the relationship between concentrations (*C*) of TSC and its oxidation signal was investigated. As shown in Figure 9, the oxidation peak current (*I*_pa_) of TSC increased linearly with *C* of TSC in the range of 25 nM to 10,000 nM, with a linear regression equation of *I*_pa_ (μA) = 0.474 *C* (μM) and a correlation coefficient (*R*) of 0.992, indicating excellent linearity. The detection limit was 25 nM, which is comparable to the values reported in Table S1.

**Figure 10:**
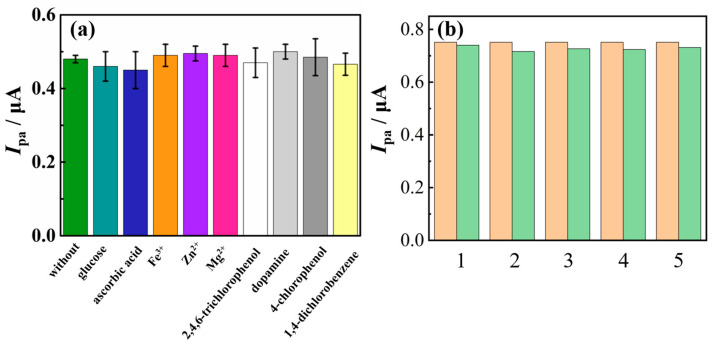
(**a**) Effect of potential interferents on oxidation peak currents of 1 μM TSC. (**b**) *I*_pa_ of 1.5 µM TSC in 0.1 M phosphate buffer solution (pH 7.0) measured 7 days prior (**left**) and 7 days later (**right**) based on Cu-BTC.

Furthermore, the anti-interference capability of the electrochemical sensor was investigated, and the effects of several common interferents on TSC detection were studied. According to Figure 10a, the glucose and ascorbic acid at 0.025 mM, as well as Fe^3+^, Zn^2+^, and Mg^2+^ at 0.05 mM, 2,4,6-trichlorophenol at 0.02 mM, dopamine at 0.02 mM, 4-chlorophenol at 0.01 mM, and 1,4-dichlorobenzene at 0.01 mM did not interfere with the detection of 1 μM TSC (peak current change less than 5%). To further investigate the anti-interference capability of nano-Cu-BTC in complex samples, tests were conducted in the presence of various interfering substances. The DPV curves showed that nano-Cu-BTC still exhibited good anti-interference capability even with the coexistence of multiple interfering substances (Figure S1, Electronic Supplementary Materials). Stability and repeatability are also important factors for evaluating the analytical performance of the obtained sensor. Five nano-Cu-BTC modified electrodes were used to measure the *I*_pa_ of 1.5 µM TSC in 0.1 M phosphate buffer solution (pH 7.0), and after being placed in ambient air at room temperature for 7 days, another set of five modified electrodes were prepared to measure the *I*_pa_ of 1.5 µM TSC in 0.1 M phosphate buffer solution (pH 7.0). The experimental results showed that the RSD of the oxidation current values obtained from five parallel running experiments were less than 5%. Furthermore, while comparing the current values obtained seven days previous to the deployment with the current values measured after a week, it was observed that the change in current was less than 5% (Figure 10b). This suggests that the electrochemical sensor exhibits excellent stability and repeatability.

To further assess the practicability of the developed electrochemical sensor, the contents of TSC in real water samples were determined. A mixture of 5 mL of filtered tap water and 5 mL of phosphate buffer solution (0.1 M, pH 7.0) was prepared. Even at very low concentrations of TSC in real water samples, there is still a good response signal on the nano-Cu-BTC (Figure S2, Electronic Supplementary Materials). The content of TSC in a series of samples was determined using the standard addition method. Each water sample was tested in triplicate, resulting in RSD values of less than 5.0% and recovery rates ranging from 96% to 108%, indicating satisfactory accuracy and application potential in real samples (Table S2, Electronic Supplementary Materials).

## 3. Materials and Methods

### 3.1. Chemicals and Instrument

Copper(II) nitrate trihydrate (Cu(NO_3_)_2_·3H_2_O), 1,3,5-benzenetricarboxylic acid (H_3_BTC), and Triclosan (TSC) standard solution (1 mg mL^−1^ in methanol) were procured from Aladdin (Shanghai Aladdin Biochemical Technology Co., Ltd., Shanghai, China). Triethylamine and N,N-dimethylformamide (DMF) were also obtained from Aladdin. All chemicals utilized in this study were of analytical grade purity and used without further purification, while ultrapure water with a resistivity of 18.2 MΩ cm was generated using a Milli-Q water purification system. Nano-Cu-BTC morphology was examined via FE-SEM (Hitachi SU8010, Hitachi, Tokyo, Japan), and its crystalline structure was analyzed by XRD (X’Pert PRO, Nalytical, Amsterdam, The Netherlands). Elemental composition and chemical states were determined using FT-IR (Equinox-55, Bruker, Karlsruhe, Germany) with KBr plates. Surface area and pore size were assessed by the BET method (BET ASAP2420-4MP, Micromeritics, Norcross, GA, USA).

### 3.2. Synthesis of Nano-Cu-BTC

The preparation of nano-Cu-BTC material follows a procedure based on a previous report [41]. Initially, 1.0 mM of H_3_BTC is dissolved in 50 mL of DMF solution with stirring until complete dissolution. Subsequently, 3.0 mM of triethylamine is introduced to the solution and stirred for 30 min. Following this, 1.0 mM of Cu(NO_3_)_2_·3H_2_O is added to the aforementioned mixed solution and stirred for an additional 30 min. The resulting mixture is then allowed to stand at room temperature for 24 h. Subsequent to this incubation period, the reaction solution is collected via centrifugation at 4000 rpm/min and washed with ethanol thrice. Finally, the precipitates obtained are vacuum dried at 60 °C for 24 h, resulting in the formation of blue nano-Cu-BTC powder.

### 3.3. Preparation of Nano-Cu-BTC Modified CPE and CPE

The procedure begins by thoroughly mixing 0.100 g of nano-Cu-BTC powder with 0.900 g of graphite powder to ensure a total mass of 1.000 g, with nano-Cu-BTC comprising 10% of the carbon paste by mass. Specifically, 0.2 mL of paraffin oil is added incrementally to the mixture, with a fixed amount added each time. Subsequently, the mixture is ground thoroughly in a mortar until it becomes uniform, resulting in the preparation of the nano-Cu-BTC-modified material. For comparison purposes, a bare carbon paste electrode (CPE) is prepared using 1.000 g of graphite powder and 0.2 mL of paraffin oil following the same procedure. Finally, both mixtures are filled and pressed into the cavity of a 3.0 mm diameter carbon paste electrode, and the surface of the electrode is polished on sulfuric acid paper until smooth for subsequent use.

### 3.4. Electrochemical Characterization

Electrochemical experiments were conducted with a CHI 660E workstation using a three-electrode system: SCE (Rosemead, CA, USA) as the reference electrode, platinum wire as the counter electrode, and nano-Cu-BTC modified CPE or CPE as the working electrode. TSC determination was carried out in a 0.1 M phosphate buffer solution (pH = 7.0) as a supporting electrolyte. DPV curves were recorded from 0.30 to 0.90 V with an oxidation peak current at 0.69 V serving as a TSC response signal. DPV parameters included 50 mV pulse amplitude, 40 ms pulse width, and 40 mV s^−1^ scan rate.

## 4. Conclusions

In summary, the study successfully synthesized a nano-Cu-BTC material MOF at room temperature using a solvent technique. The incorporation of triethylamine enabled the control of ligand deprotonation, resulting in the production of homogeneous Cu-BTC nanoparticles. Additionally, the study explored the electrochemical interaction of nano-Cu-BTC with triclosan and developed an effective electrochemical sensor for triclosan by leveraging the signal amplification effect of nano-Cu-BTC. The experimental conditions were optimized using response surface methodology, resulting in a straightforward, rapid, and highly sensitive triclosan detection method with a linear range of 25–10,000 nM and a detection limit of 25 nM. The findings highlight the significant potential of metal–organic frameworks, particularly nano-Cu-BTC, in enhancing the active sites and reactive surface area of electrochemical sensors, thus advancing environmental monitoring and public health protection.

## Figures and Tables

**Table 1:** Experimental design provided by Design Expert software and corresponding experimental outcomes.

No.	Enrichment Time (s)	Nano-Cu-BTC Modification Amount (%)	Enrichment Potential (V)	*I*_pa_ (μA)
1	300	8	0.4	0.099
2	180	8	0.1	0.100
3	300	4	0.1	0.042
4	180	4	−0.2	0.063
5	180	8	0.1	0.100
6	60	8	0.4	0.066
7	300	12	0.1	0.072
8	180	12	−0.2	0.044
9	60	8	−0.2	0.043
10	300	8	−0.2	0.081
11	180	8	0.1	0.100
12	180	8	0.1	0.100
13	60	4	0.1	0.090
14	180	8	0.1	0.100
15	180	4	0.4	0.076
16	180	12	0.4	0.052
17	60	12	0.1	0.041

## Data Availability

Data will be made available upon reasonable request.

## Supplementary Materials

The following supporting information can be downloaded at: https://www.mdpi.com/article/10.3390/molecules29143298/s1, Figure S1: The interference test in the coexistence of multiple interfering substances. a: The DPV curve of 1 μM TSC without interfering substances; b: the DPV curve of 1 μM TSC with 0.025 mM glucose and 0.025 mM ascorbic acid; c: the DPV curve of 1 μM TSC with 0.05 mM Fe^3+^, Zn^2+^, and Mg^2+^; d: the DPV curve of 1 μM TSC with 0.02 mM 2,4,6-trichlorophenol and dopamine, and 0.01 mM 4-chlorophenol and 1,4-dichlorobenzene; Figure S2: DPV curves of the nano-Cu-BTC in a water sample at the optimum test condition. A: Blank control, B: water sample; Table S1: Comparison of Electrochemical Methods for TSC; Table S2: Application of nano Cu-BTC in the detection of TSC in real water samples. Refs. [42,43,44,45,46,47] are cited in Supplementary Materials.
