# Supplementary material for: User-Friendly and Responsive Electrochemical Detection Approach for Triclosan by Nano-Metal–Organic Framework
Source: Molecules. 2024 Jul 12;29(14):3298. doi: 10.3390/molecules29143298 (PMC11279189; doi:10.3390/molecules29143298)
Supplement: Supplementary file 1 [file molecules-29-03298-s001.zip › molecules-3041505-supplementary.pdf]

Supporting Information

User-Friendly and Responsive Electrochemical Detection Approach for Triclosan by Nano-Metal–Organic Framework

Xiaoyu Li[1, 2, *], Gaocheng Zhang[3], Zareen Zuhra[1], and Shengxiang Wang[3,*]

[1] School of Bioengineering and Health, Wuhan Textile University, Wuhan 430200, China
[2] State Key Laboratory of New Textile Materials and Advanced Processing Technologies, Wuhan Textile University, Wuhan 430200, China
[3] School of Mathematical and Physical Sciences, Wuhan Textile University, Wuhan 430200, China
* Correspondence: xyli@wtu.edu.cn (X.L.); shxwang@wtu.edu.cn (S.W.)

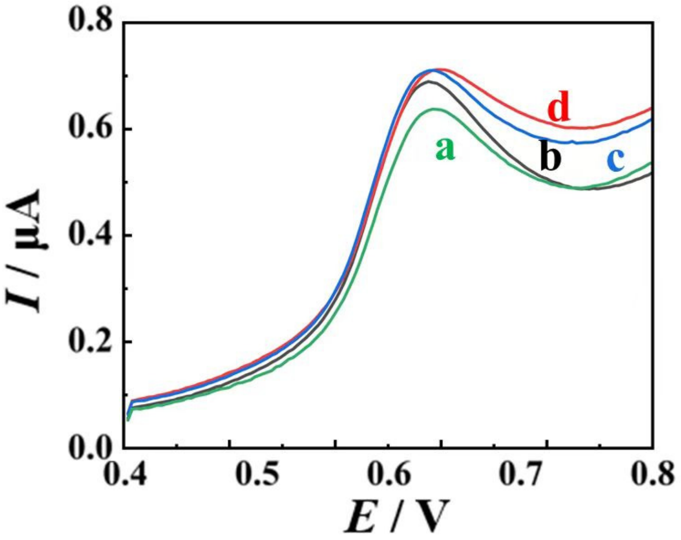

Figure S1. The interference test in coexisting multiple interfering substances. a: the DPV curve of 1 μM TSC without interfering substances; b: the DPV curve of 1 μM TSC with 0.025 mM glucose and 0.025 mM ascorbic acid; c: the DPV curve of 1 μM TSC with 0.05 mM Fe^{3+}, Zn^{2+}, and Mg^{2+}; d: the DPV curve of 1 μM TSC with 0.02 mM 2,4,6-trichlorophenol and dopamine, 0.01 mM 4-chlorophenol and 1,4-dichlorobenzene.

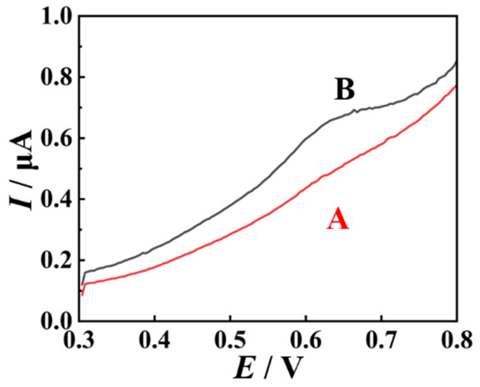

Figure S2. DPV curves of the nano Cu-BTC in water sample at the optimum test condition. A: blank control, B: water sample.

Table S1. Comparison of Electrochemical Methods for TSC

Sensor	Linearity range	Detection limit （nM）	Reference
2-Hp-b-CD	1×10^{-8} M - 1×10^{-4} M	9.78	1
β-CD-MW-CNT	1×10^{-8} M - 1×10^{-4} M	9.87	2
ZIF-11/RHAC	1×10^{-6} M - 8×10^{-6} M	76	3
N-GQD-CS/GCE	0.05×10^{-6} M - 8.0×10^{-6} M	14	4
GQD-chitosan/GCE	0.10×10^{-6} M - 10.0×10^{-6} M	30	5
CPT-BDD	3.5×10^{-8} M - 3.5×10^{-6} M	7.9	6
Nano Cu-BTC	25×10^{-9} M - 1×10^{-6} M	25	This work

Note: 2-Hp-b-CD: hydrophilic 2-hydroxypropyl β-cyclodextrin; β-CD-MW-CNT: β-cyclodextrin modified multi-walled carbon nanotubes; ZIF-11/RHAC: ZIF-11/activated carbon derived from the rice husk modified electrode ; N-GQD-CS/GCE: nitrogen-doped graphene quantum dots supported in chitosan/ glass carbon electrode; GQD-chitosan/GCE: graphene quantum dots supported in chitosan/ glass carbon electrode; CPT-BDD: cathodically pretreated boron-doped diamond electrode in the presence of cationic surfactant.

Table S2. Application of nano Cu-BTC in the detection of TSC in real water samples.

Sample	detected / (nM)	added / (nM)	presence / (nM)	Recovery (%)	RSD (%)
S1	30	30	62	107	4.7
S2	-	50	48	96	2.8
S3	50	50	102	104	3.6

Reference

1.Safwat, N.; Mahmoud, A. M.; Abdel-Ghany, M. F.; Ayad, M. F. In Situ Monitoring of Triclosan in Environmental Water with Subnanomolar Detection Limits Using Eco-Friendly Electrochemical Sensors Modified with Cyclodextrins. *Environmental Science: Processes & Impacts* **2021**, *23* (3), 457–466. DOI 10.1039/d0em00387e

2.Safwat, N.; Mahmoud, A. M.; Abdel-Ghany, M. F.; Ayad, M. F. Eco-Friendly Monitoring of Triclosan as an Emerging Antimicrobial Environmental Contaminant Utilizing Electrochemical Sensors Modified with CNTs Nanocomposite Transducer Layer. *BMC chemistry* **2023**, *17* (1), 170. DOI 10.1186/s13065-023-01092-0

3.Luyen, N. D.; Toan, T. T. T.; Trang, H. T.; Nguyen, V. T.; Son, L. V. T.; Thanh, T. S.; Thanh, N. M.; Quy, P. T.; Khieu, D. Q. Electrochemical Determination of Triclosan Using ZIF-11/Activated Carbon Derived from the Rice Husk Modified Electrode. *Journal of Nanomaterials* **2021**, *2021*, 1–14. DOI 10.1155/2021/8486962

4. Santana, E. R.; Martins, E. C.; Spinelli, A. Electrode Modified with Nitrogen-Doped Graphene Quantum Dots Supported in Chitosan for Triclocarban Monitoring. *Microchemical Journal* **2021**, *167*, 106297. DOI 10.1016/j.microc.2021.106297
5. Santana, E. R.; Spinelli, A. Electrode Modified with Graphene Quantum Dots Supported in Chitosan for Electrochemical Methods and Non-Linear Deconvolution of Spectra for Spectrometric Methods: Approaches for Simultaneous Determination of Triclosan and Methylparaben. *Microchimica Acta* **2020**, *187*, 1–12. DOI 10.1007/s00604-020-04225-7
6. Pınar, P. T.; Allahverdiyeva, S.; Yardım, Y.; Şentürk, Z. Voltammetric Sensing of Dinitrophenolic Herbicide Dinoterb on Cathodically Pretreated Boron-Doped Diamond Electrode in the Presence of Cationic Surfactant. *Microchemical Journal* **2020**, *155*, 104772. DOI 10.1016/j.microc.2020.104772
